# Knowledge, Attitudes, and Practice of Guided Dentistry Among Dental Personnel of North India: A Cross-Sectional Study

**DOI:** 10.7759/cureus.71991

**Published:** 2024-10-21

**Authors:** Shikha Jaiswal, Ayushi Sharma, Sachin Gupta, Shreshtha Sharma, Suveksha Sahay, Shivali Tyagi

**Affiliations:** 1 Conservative Dentistry and Endodontics, Subharti Dental College and Hospital, Meerut, IND

**Keywords:** computer-assisted dynamic navigation, guided dentistry, guided endodontics, static guided endodontics, survey.

## Abstract

Objective

The aim of this study was to assess the knowledge and awareness of guided dentistry among various dental professionals.

Materials and methods

The study was carried out as a cross-sectional descriptive observational study among dental professionals (students, practitioners, and teaching faculty in academic institutions) in North India. A web-based electronic survey using Google Forms was designed to obtain the responses of participants toward awareness of guided dentistry and its applications in dentistry. A total of 2200 responses were collected with a response rate of 71.6%. The stratified sampling method was used to allocate participants into different strata.

Results

Among different professionals, teaching faculty in academics had the highest level of awareness about the concept of guided dentistry. Academic lectures and dental workshops were the major sources of information about this concept. A significantly higher level of awareness regarding the Dynamic Navigation System was there among academicians as compared to other dental professionals (p<0.01). Expensive nature and limited learning opportunities were cited as the major cause for the limited practice of this procedure.

Conclusions

Within the limitation, the majority of dental professionals in North India had a decent awareness of guided dentistry but a limited clinical approach owing to strata-specific factors. Academic faculty, dental students, and dental practitioners had better awareness and positive attitudes toward its frequent clinical approach.

## Introduction

The field of medicine and dentistry has benefited enormously in various aspects owing to the incorporation of digital dentistry be it diagnosis or treatment planning. Dental navigation or guided dentistry is one such application of digital dentistry that has shown immense potential in development and implementation in dentistry [1]. In the United States, navigation systems were first used in dentistry in the year 2000 to ease the placement of dental implants and direct the drill by creating a surgical template using precision software, cone beam computed tomography (CBCT), and 3-dimensional (3D) scanning pictures [2], and then this technique was termed guided dentistry. Many in vitro studies [3-5] show that this technique proved to be accurate, expeditious, and operator-independent. Recent clinical case reports show the successful application of this novel technique in various fields of dentistry such as Endodontics [3], Implantology [6], Surgery [7], Orthodontics [8], etc., with its biggest advantage being its predictable outcome, though requiring learning curve with extensive clinical training. For widespread understanding and implementation, it is important that dental professionals have a thorough knowledge and understanding of the concept of guided dentistry [9]. Hence, this study was undertaken with the intent of assessing the knowledge, awareness, and practices regarding the use of guided dentistry among dental professionals in North India.

## Materials and methods

Setting and design

A web-based electronic survey was designed for this research. A Google form consisting of 15 questions was formulated and reviewed by four independent experts from the field (see Appendices). The final questionnaire was prepared after reaching a common consensus, which was further pilot-tested on a small sample of the intended respondents. The form was sent to participants through WhatsApp and Gmail, which they access and reply to using their mobile, computer/tablets, etc. The data was collected from 2200 participants and responses were generated between September 2022 to December 2022 with a response rate of 71.6%. The inclusion criteria for the respondents included final year BDS (Bachelor of Dental Surgery) students and interns in the undergraduate (UG) category, postgraduate (PG) students from all specialties, and academicians and dental practitioners of varied experience. The exclusion criteria were 1st year, 2nd year, and 3rd year BDS students. Participation was voluntary and written informed consent was obtained in the Google form once the respondents had read and understood the study information sheet.

Sample size calculation

The sample size was determined using the formula: N = z2pq/L2 with a Type I error rate set at 0.05 and the power of the study at 80%. 2150 respondents were required to satisfy the requirement for analysis of a cross-sectional study. The sample size was increased to account for potential non-responses due to participant ineligibility and incomplete response. Stratified sampling was used to allocate participants into three subgroups i.e.; dental students (n=1880), teaching faculty in academic institutions (n=70), and dental practitioners (n= 250).

Questionnaire development and testing

The questionnaire developed was in English language and consisted of three parts: Part A - Knowledge and Awareness, Part B - Technical Details and Applications, and Part C - Attitude and Learning Resource. Part A questions were related to familiarity with the concept of guided dentistry and its uses, ideas on how Guided Dentistry can be incorporated into a dental practice, and have the respondents attended any training program related to guided dentistry, etc. Part B was more focused on the application of Guided dentistry, e.g., its application in the field of endodontics (Guided Endodontics), will the application of guided dentistry enhance their clinical practice, why guided dentistry is a less-practised modality among dental personnel, etc. Part C included questions about the intent of learning and attitude of respondents with respect to their thoughts regarding inclusion of guided dentistry in undergraduate and postgraduate curriculum, preference of their source to learn more about guided dentistry, etc.

Demographic details

The respondents were mostly females (64.9%), the students /academicians were mostly from the private sector (59%) as compared to the government sector (41%), the practitioners were majorly from urban/semi-urban (73.8%) practice setting as compared to rural settings (26.2%).

Statistical analysis

Data were analyzed using the Statistical Package for the Social Sciences, software version 22.0 for Windows (IBM Corp., Armonk, USA). Descriptive analysis was used to define the characteristics of the variables using numbers and percentages. To establish an association between groups, a One Way Anova - F test was performed, and statistical significance was recognized when p < 0.01.

## Results

A total of 2200 responses were collected. The participants were dental students (n=1880), teaching faculty in academic institutions (n=70), and dental practitioners (n=250) from various specialties of dentistry with varied degrees of experience. Table 1 summarizes that among different professionals, teaching faculty in academics had the highest level of awareness about the concept of guided dentistry (n=60; 85.71 %). Academic lectures and dental workshops were the major sources of information amongst all others. As compared to dental practitioners, academicians had significantly better knowledge about prerequisites for guided dentistry. (p<0.01) Among the components of guided dentistry, there was significantly less (p<0.001) awareness of guided drills and 3-dimensional printers. All the teaching faculty in academic institutions were significantly more aware of the Dynamic Navigation System compared to other dental professionals (p<0.01). Among the various applications guided dentistry was more significantly (p<0.01) being utilized for implantology and endodontics as compared to others.

**Table 1:** Table establishing relationships between categorical variables (i.e., options and responses)-I *Indicated statistically significant differences (p < 0.01).

S.NO	QUESTIONS ASKED	OPTIONS	FREQUENCIES (%)			P-VALUE (ONE WAY ANOVA – F TEST)
			ACADEMICIANS (N=70)	DENTAL PRACTITIONERS (N=250)	DENTAL STUDENTS (N=1880)	
1	Are you familiar with the concept of guided dentistry?	Yes	60 (85.71%)	210 (84%)	1540 (81.92 %)	p=.0014*
		No	10 (14.29%)	40 (16%)	340 (18.09%)	
2	Do you have any idea of how guided dentistry can be incorporated in dental practice?	Yes	60 (85.71%)	190 (76%)	1620 (86.17 %)	p=.0024*
		No	10 (14.29%)	60 (24%)	260 (13.83%)	
3	What was the information source about guided dentistry application?	Academic lectures	10 (14.29%)	100 (40%)	720 (38.30%)	p=.0002*
		Dental workshop	50 (71.42%)	10 (28%)	760 (40.43 %)	
		Not yet aware	10 (14.29%)	40 (16%)	330 (17.55%)	
		electronic media	00 (00%)	30 (12%)	60 (3.19%)	
		Via colleagues	00 (00%)	100 (40%)	10 (.53%)	
4	Have you attended any training programs on guided dentistry?	Yes	20 (28.57%)	50 (20%)	410 (21.81%)	p=.0003*
		No	50 (71.42%)	200 (80%)	1470 (78.19%)	
5	Enlist the prerequisites for guided dentistry					
5 (a)	Cone beam computed tomography	1	50 (71.42%)	190 (76%)	1280 (68.09%)	p=.0005*
		0	20 (28.57%)	60 (24%)	600 (31.91%)	
5 (b)	Intraoral and extraoral scanners	1	50 (71.42%)	190 (76%)	1070 (56.91%)	p=.0029*
		0	20 (28.57%)	60 (24%)	810 (43.09%)	
5(c)	Specialized software and drills	1	40 (57.14%)	16 (64%)	1000 (53.19 %)	p=.0033*
		0	30 (42.86%)	90 (36%)	880 (46.81 %)	
5(d)	3-dimensional printing	1	40 (57.14%)	130 (52%)	780 (41.49 %)	p=.0011*
		0	30 (42.86%)	120 (48%)	1100 (58.51 %)	
6	Which type of guided modality has a clinical real-time interface display and guides the drill into the targeted position through the prefixed trace according to the preoperative planning software.	Blended navigation system	00 (00%)	40 (16%)	180 (9.57 %)	p=.0004*
		Dynamic navigation system	50 (71.42%)	140 (56%)	1230 (65.43 %)	
		None	00 (00%)	60 (24%)	160 (8.51 %)	
		Static navigation system	20 (28.57%)	10 (4%)	310 (16.49 %)	
7	Have you performed guided dentistry in your practice? If yes, in which clinical scenario?	Endodontics	20 (28.57%)	40 (16%)	80 (4.26 %)	p=.0009*
		Implantology	30 (42.86%)	50 (20%)	110 (5.86 %)	
		Orthodontics	10 (14.29%)	00 (00%)	10 (.53%)	
		Surgery	00 (00%)	10 (4%)	60 (3.19 %)	
		Not performed	10 (14.29%)	150 (60%)	1620 (86.16 %)	

As shown in Table 2, most of the respondents were unaware of the use of guided endodontics for fiber post removal in comparison to pulp canal obliteration cases, in cases of retreatment and missed canals, and in cases of endodontic surgery and this difference was statically significant (p<0.01). Despite of lack of awareness about the application of guided dentistry, most respondents agreed that it will enhance their clinical practice (n=2120; 96.3%). The major reason cited behind the limited practice of guided dentistry was the expensive nature of the procedure and anticipation of technical difficulty (p<0.01).

**Table 2:** Table establishing relationships between categorical variables, (i.e., options and responses)-II *Indicated statistically significant differences (p < 0.01).

S.NO	QUESTIONS ASKED	OPTIONS	FREQUENCIES (%)			P-VALUE (ONE WAY ANOVA – F TEST)
			ACADEMICIANS (N=70)	DENTAL PRACTITIONERS (N=250)	DENTAL STUDENTS (N=1880)	
8	Application of Guided dentistry in the field of endodontics (Guided Endodontics).					
8 (a)	For treatment of pulp canal obliteration	1	50 (71.42%)	170 (68%)	1210 (64.36%)	p=.0008*
		0	20 (28.57%)	80 (32%)	670 (35.64%)	
8(b)	For retreatment	1	40 (57.14%)	150 (60%)	750 (39.89%)	p.0004*
		0	30 (42.86%)	100 (40%)	1130 (60.11%)	
8(c)	For fiber post removal	1	20 (28.57%)	130 (52%)	640 (34.04%)	p=.0002*
		0	50 (71.42%)	120 (48%)	1240 (65.96%)	
8(d)	In cases of endodontic surgery	1	50 (71.42%)	170 (68%)	1080 (57.45%)	p=.0001*
		0	20 (28.57%)	80 (32%)	800 (42.55%)	
8(e)	In cases of missed canals	1	30 (42.86%)	170 (68%)	880 (46.81%)	p=.0008*
		0	40 (57.14%)	80 (32%)	1000 (53.19%)	
9	Do you think application of guided dentistry will enhance your clinical practice?	Yes	60 (85.71%)	230 (92%)	1830 (97.34%)	p=.0012*
		No	10 (14.29%)	20 (08%)	50 (2.66%)	
10	Why do you think guided dentistry is less practiced modality among dental personnels?					
10(a)	Lack of knowledge	1	50 (71.42%)	170 (68%)	1240 (65.96%)	p=.0013*
		0	20 (28.57%)	80 (32%)	640 (34.04%)	
10(b)	Expensive	1	60 (85.71%)	180 (72%)	1180 (62.77%)	p=.0001*
		0	10 (14.29%)	70 (28%)	700 (37.23 %)	
10(c)	Lack of armamentarium	1	50 (71.42%)	140 (56%)	850 (45.21 %)	p=.0006*
		0	20 (28.57%)	110 (44%)	1030 (54.79%)	
10(d)	Technical dexterity	1	20 (28.57%)	90 (36%)	620 (32.98%)	p=.0004*
		0	50 (71.43%)	160 (64%)	1260 (67.02%)	

As shown in Table 3, the majority of the respondents reported that guided dentistry should be included in both undergraduate (UG) and postgraduate (PG) curricula (p<0.001). The majority of the subjects would like themselves to be treated with this modality if needed and wanted to learn more about the same in the future (n=1910; 86.81%). Responses to the minimally invasive method (n=50; 71.42%) and its precise and accurate nature (n=50; 71%) were reported as the most significant advantages of guided dentistry when compared to the advantage of the decrease in duration of treatment amongst various groups (p<0.01). The majority of the respondents did not attend any training program on guided dentistry. Among the 21.81% of dental students who attended the training program, 20.7% were postgraduate students, while only 1.11% were undergraduate interns. This difference is likely due to the greater application of guided technique among postgraduate students. On the other hand, 20% of dental practitioners attended the training program. Of these, only 2% held undergraduate degrees and were currently practicing implantology, while the remaining 18% were practitioners with postgraduate degrees. Among them, 15.6% were oral implantologists (including oral surgeons and periodontists), and the remaining 2.4% were endodontists and prosthodontists. 

**Table 3:** Table establishing relationships between categorical variables (i.e. options and responses)-III *Indicated statistically significant differences (p < 0.01).

S.NO	QUESTIONS ASKED	OPTIONS	FREQUENCIES (%)			P-VALUE (ONE WAY ANOVA – F TEST)
			ACADEMICIANS (N=70)	DENTAL PRACTITIONERS (N=250)	DENTAL STUDENTS (N=1880)	
11	Do you think guided dentistry should be included in undergraduate/postgraduate curriculum?	Undergraduate	00 (00%)	00 (00%)	270 (14.36 %)	p=.0001*
		Postgraduate	30 (42.86%)	70 (28%)	350 (18.62%)	
		Both	40 (57.14%)	180 (72%)	1220 (64.89%)	
		Not required	00 (00%)	00 (00%)	40 (2.13 %)	
12	What according to you are advantages of using guided dentistry?					
12(a)	Reduced iatrogenic errors	1	10 (14.29%)	60 (24%)	750 (39.89%)	p=.0002*
		0	60 (85.71%)	190 (76%)	1130 (60.11%)	
12(b)	Decrease the duration of treatment	1	30 (42.86%)	110 (44%)	850 (45.21%)	p=.0004*
		0	40 (57.14%)	140 (56%)	1030 (54.79%)	
12(c)	Minimally invasive method	1	50 (71.42%)	160 (64%)	1030 (54.79%)	p=.0028*
		0	20 (28.57%)	90 (36%)	850 (45.21%)	
12(d)	Precise and accurate	1	50 (71.42%)	240 (96%)	1290 (68.62%)	p=.0021*
		0	20 (28.57%)	10 (4%)	590 (31.38%)	
13	Would you prefer to get treated with guided dentistry modality, if needed?	Yes	60 (85.71%)	200 (80%)	1300 (69.15%)	p=.0016*
		No	10 (14.29%)	50 (20%)	580 (30.86%)	
14	Would you like to learn about guided dentistry in future?	Yes	60 (85.71%)	210 (84%)	1640 (87.23%)	p=.0017*
		No	10 (14.29%)	40 (16%)	240 (12.77%)	
15	Which among the following options would you choose as information source about guided dentistry application?					
15(a)	Lectures	1	30 (42.86%)	110 (78.57%)	720 (38.30%)	p=.0003*
		0	40 (57.14%)	140 (21.43%)	1160 (61.70%)	
15(b)	Hands-on workshop	1	60 (85.71%)	230 (92%)	1700 (90.43%)	p=.0002*
		0	10 (14.29%)	20 (08%)	180 (9.57%)	
15(c)	Electronic media (YouTube, Facebook, etc.)	1	30 (42.86%)	60 (24%)	1550 (82.45%)	p=.0004*
		0	40 (57.14%)	190 (76%)	330 (17.55%)	
15(d)	Not interested	1	10 (14.29%)	10 (4%)	50 (2.66%)	p=.0005*
		0	60 (85.71%)	240 (96%)	1830 (97.34%)	

A larger proportion of academicians (28.57%) attended the training program compared to dental practitioners and students. Of these, 25.67% were implantologists, while the remaining 2.9% were endodontists. Regarding the preferred information source for guided dentistry, hands-on workshops were the top choice (n=1990; 90.4%) for learning about the field, followed by electronic media. This preference is due to the workshops offering practical, first-hand experience and addressing technical challenges before applying the techniques in a clinical setting. Of the 85.71% of academicians, 65.82% were oral implantologists (including oral surgeons and periodontists), followed by 15.2% who were endodontists and prosthodontists, with the remaining 4.69% specializing in combined disciplines. Among the 92% of dental practitioners, 12.8% held undergraduate degrees, while the remaining 79.2% were postgraduates. Of these, 64.8% were oral surgeons and implantologists, while the remaining 14.4% represented combined specialties, including endodontists, prosthodontists, and orthodontists. Among the 90.43% of dental students, 82.57% of postgraduate students preferred hands-on workshops for learning guided dentistry, likely due to their greater awareness and knowledge compared to only 7.86% of undergraduate students. This difference may be attributed to undergraduates' lack of awareness and their belief that guided techniques would be more useful at later stages of their practice, unlike postgraduates.

## Discussion

Guided dentistry is an umbrella term that has wide applications like guided implantology, guided surgery, guided biopsy, guided endodontics, and guided orthodontics. It can be categorized into Static and Dynamic types [1]. In static technique, Cone beam computed tomography is merged with an optical impression to create a platform for the design of a virtual drill path, following which templates are fabricated through 3-dimensional printing [10]. Literature shows evidence regarding the efficacy of static and dynamic guided techniques *in vivo*. Connert T et al [3,11] when comparing guided endodontics to traditional access cavity concluded that the guided approach showed less dentin loss and was not much influenced by the experience of the operator. Also, Valdec S et al [4] observed that there is increased precision and predictability, better access to deep-seated locations in complex anatomical regions like the tongue, and a shorter duration of surgical intervention when the guided biopsy technique was used as compared to the conventional technique.

Despite numerous advantages, the static technique is limited by issues of static templates, difficulties in rubber dam placement, dentin debris removal, and restricted mouth opening. These issues can be resolved by adapting dynamic navigation which addresses spatial constraints and offers flexibility for intraoperative adjustments thus improving treatment outcomes. Dr. David Burgress very rightly compared the dynamic technique with the Global Positioning System installed in our automobiles [12] as it uses an optical positioning device controlled by a dedicated computerized interface [11]. This real-time interface displays the drill into the targeted position [13].

According to this study, teaching faculty in academic institutions showed better awareness and knowledge about the concept and technique of guided dentistry as compared to dental practitioners. This result could be possibly due to the fact that the work profile of academicians is related more to research and knowledge about the latest innovations in their fields. However, dental practitioners who do not attend education programs and workshops lack this knowledge. This highlights the importance of dental education program's efficacy in advanced techniques for professional development especially for practitioners. Dental workshops and educational lectures were the primary sources of information for the personnel. This could be due to their structured format, hands-on learning opportunities, and direct interaction with experts, which make them effective in staying updated with the latest advancements in the field. Since electronic media also formed one of the main sources of information, this not only implies the strong impact of electronic media in all fields including education but also gives a cue that it can be used as a strong tool to promote guided dentistry. In addition, cases done through guided dentistry should be shared by dental personnel on electronic media platforms, this would garner not only attention and curiosity but also inspire fellow dentists to adopt this approach more commonly in their practice.

Dental professionals were more aware of static rather than dynamic system of guided dentistry, which may be because the dynamic system is relatively new and there are very few practitioners who are practicing this system. The results of this survey showed that the field of endodontics and implantology found more application with this guided technique compared to the other fields. This can be due to a greater number of patients undergoing implants or complex endodontic procedures. Regarding the application of guided dentistry in the field of endodontics (Guided Endodontics), responses to fiber post-removal differed significantly. This could be possibly due to fewer cases of fiber post-removal performed and the complexity and iatrogenic errors associated with the same. This technique provides more reliable outcomes, reduces working time, and eases the work of clinicians so it may be a promising method for the endodontic or surgical treatment of complex cases. Most respondents said that guided dentistry could enhance clinical practice but the expensive nature of the treatment was the main deterrent in its decreased usage in clinical practice. This was in consensus with one of the previous studies that was exclusively done for guided endodontics, which also stated that the expensive nature is the main hazard for the use of guided endodontics in clinics [14].

There was a common consensus among all for the inclusion of guided dentistry in undergraduate and postgraduate curricula which would familiarize the students with the technique and pave the way for further learning in the future. This emphasizes the need for constant updating of the academic curriculum and the need for designing new policies and revising the current curriculum. The majority of the respondents would like themselves to be treated with this modality if needed and were keen to attend the dental workshop and educational program to update their knowledge as the majority of the participants had no previous experience with the guided dentistry procedure. The majority of respondents wanted to learn more about the same in the future as they thought it to be a minimally invasive method [15], precise and accurate [16]. This finding highlights the need to undertake surveys, dental education programs, seminars, or hands-on courses to shift dentistry towards digital technology and to increase dentists’ expertise and awareness.

The results and outcome of this survey may be limited by the fact that any survey faces a risk of of low response rate, and dishonest or inaccurate answers (due to misinterpretation of questions)[17]. Additionally, survey fatigue may affect the quality and accuracy of the response. Although in anonymous surveys like these, the risk is usually lower, however, it does not disappear entirely [18].

## Conclusions

Within the limitations of this study, it can be concluded that although guided dentistry offers a predictable and minimally invasive treatment option, improves the prognosis of teeth, and prevents complications in complex cases, dental practitioners lack awareness regarding the technique, usage, and application of guided dentistry. Therefore, there’s a need for a greater number of hands-on workshops to train the dentist regarding the practice of guided dentistry. Also, since the expensive nature of the procedure is a deterrent for clinical practice, methods should be devised to make the treatment more cost-effective. Also, dental workshops and educational lectures would be excellent learning tools to popularize this procedure. The clinical significance of this survey was to concentrate on the difficulties encountered in clinically practising this novel approach and, after understanding them, focus on reforms to increase its utilization among various professionals.

## Appendices

Questions asked to the dental professionals using Google Forms

Q1-Are you familiar with the concept of Guided dentistry and its uses?

A.    Yes

B.    No

Q2-Do you have any idea of how GD can be incorporated in dental practice?

A.    Yes

B.    No

Q3-Have you attended any training programs on Guided dentistry.

A.    Yes

B.    No

Q4-Have you performed guided dentistry in your practice, if yes, in which clinical scenario.

A.    Guided Implantology

B.    Guided Implantology and Guided Endodontics

C.    Guided Orthodontics and Guided biopsy

D.    Not performed

Q5-Application of Guided dentistry in the field of endodontics (Guided Endodontics).

A.    Pulp canal obliteration

B.    Retreatment

C.    Post space preparation

D.    Endodontic surgery

E.     All of the above

Q6-Do you think application of guided dentistry will enhance your clinical practise?

A.    Yes

B.    No

Q7-Will you recommend fellow practitioners to implement guided dentistry in their clinical practice.

A.    Yes

B.    No

C.    Maybe

Q8-Which sector of health care do you think will be the first to commercialize Guided dentistry?

A.    Public health centres

B.    Private clinics

C.    Specialized clinics

D.    University hospital

Q9-Why do you think guided dentistry is less practised modality among dental personnels?

A.    Lack of knowledge

B.    Expensive

C.    Lack of armamentarium.

D.    Technical dexterity

Q10-Enlist the prerequisites for guided dentistry-

A.    CBCT

B.    Intraoral and Extraoral scanners

C.    Specialized software’s and drills

D.    3D printing

E.     All of the above

Q11- Do you think this survey motivates you to know more about guided dentistry?

A.    Yes

B.    No

Q12-Would you like to learn about guided dentistry in future?

A.    Yes

B.    Not sure

C.    Maybe

Q13-Do you think guided dentistry should be included in undergraduate/postgraduate curriculum?

A.    Yes

B.    No

Q14-Information source about guided dentistry application?

A.    Academic lectures

B.    Dental Workshops

B.    Electronic media (Facebook, LinkedIn)

D.    Via colleagues

Q15-What according to you are advantages of using guided dentistry?

A.    Reduce iatrogenic errors

B.    Decrease the duration of treatment

C.    Minimally invasive method

D.    All of the above
